# Time to first treatment and P53 dysfunction in chronic lymphocytic leukaemia: results of the O-CLL1 study in early stage patients

**DOI:** 10.1038/s41598-020-75364-3

**Published:** 2020-10-28

**Authors:** Paola Monti, Marta Lionetti, Giuseppa De Luca, Paola Menichini, Anna Grazia Recchia, Serena Matis, Monica Colombo, Sonia Fabris, Andrea Speciale, Marzia Barbieri, Massimo Gentile, Simonetta Zupo, Mariella Dono, Adalberto Ibatici, Antonino Neri, Manlio Ferrarini, Franco Fais, Gilberto Fronza, Giovanna Cutrona, Fortunato Morabito

**Affiliations:** 1 Mutagenesis and Cancer Prevention Unit, IRCCS Ospedale Policlinico San Martino, 16132 Genoa, Italy; 2 Department of Oncology and Hemato-Oncology, University of Milan, 20122 Milan, Italy; 3 Molecular Diagnostic Unit, IRCCS Ospedale Policlinico San Martino, 16132 Genoa, Italy; 4 Biotechnology Research Unit, Aprigliano, A.O./ASP of Cosenza, 87100 Cosenza, Italy; 5 Molecular Pathology Unit, IRCCS Ospedale Policlinico San Martino, Genoa, Italy; 6 Hematology Unit, Fondazione IRCCS Ca’ Granda, Ospedale Maggiore Policlinico, 20122 Milan, Italy; 7 Hematology Unit, Department of Onco-Hematology, A.O. of Cosenza, 87100 Cosenza, Italy; 8 Hematology Unit and Bone Marrow Transplantation, IRCCS Ospedale Policlinico San Martino, Genoa, Italy; 9 Department of Experimental Medicine, University of Genoa, 16132 Genoa, Italy; 10 Department of Hematology and Bone Marrow Transplant Unit, Augusta Victoria Hospital, Jerusalem, Israel

**Keywords:** Preclinical research, Medical research, Molecular medicine

## Abstract

Chronic lymphocytic leukaemia (CLL) is characterised by a heterogeneous clinical course. Such heterogeneity is associated with a number of markers, including *TP53* gene inactivation. While *TP53* gene alterations determine resistance to chemotherapy, it is not clear whether they can influence early disease progression. To clarify this issue, *TP53* mutations and deletions of the corresponding locus [del(17p)] were evaluated in 469 cases from the O-CLL1 observational study that recruited a cohort of clinically and molecularly characterised Binet stage A patients. Twenty-four cases harboured somatic *TP53* mutations [accompanied by del(17p) in 9 cases], 2 patients had del(17p) only, and 5 patients had *TP53* germ-line variants. While del(17p) with or without *TP53* mutations was capable of significantly predicting the time to first treatment, a reliable measure of disease progression, *TP53* mutations were not. This was true for cases with high or low variant allele frequency. The lack of predictive ability was independent of the functional features of the mutant P53 protein in terms of transactivation and dominant negative potential. *TP53* mutations alone were more frequent in patients with mutated IGHV genes, whereas del(17p) was associated with the presence of adverse prognostic factors, including CD38 positivity, unmutated-IGHV gene status, and NOTCH1 mutations.

## Introduction

Decades of biological and molecular studies have provided extensive information for the understanding of chronic lymphocytic leukaemia (CLL), although many questions remain unanswered and the disease is still considered to be virtually incurable^1^. Despite the morphological and phenotypic homogeneity of CLL cells, the clinical course of the disease is highly heterogeneous ranging from rapid disease progression, requiring early treatment, to decades of survival with no treatment need^2^. This clinical course probably reflects the molecular heterogeneity of the disease. Over 80% of CLL cases harbour karyotype aberrations at diagnosis, the most frequent being partial deletions at 13q (~ 55%), 11q (~ 15%), 17p (~ 8%), and gain of chromosome 12 (~ 15%)^3^. In addition, specific gene mutations (e.g. *TP53*, *SF3B1*, *BIRC3*, *NOTCH1*, *ATM*) have been reported^4^. The incidence of *TP53* tumour suppressor gene mutations is low at diagnosis (~ 5 to 7%), but it rises as the disease progresses and reaches approximately 40% in refractory CLL^5–9^. The European Research Initiative on CLL (ERIC) group recommends *TP53* mutational screening for all patients before therapy start to avoid treatment protocols that are ineffective in patients with *TP53* alterations^10^. Furthermore, since mutational status of the immunoglobulin heavy-chain variable region gene (IGHV) and complex karyotype are relevant to improve risk stratification^11^, Baliakas et al.^12^, have recently incorporated these two markers, and *TP53* mutations into a novel prognostic model.

The *TP53* tumour suppressor gene encodes a tetrameric transcription factor that controls different pathways, through its ability to transactivate a plethora of downstream effector genes, many of which are relevant for carcinogenesis^13^. Disruption of the P53 protein pathway, which may involve different mechanisms, is highly selected in most tumour types and is frequently due to alterations of both alleles, e.g. deletion of one allele [del(17p)] and mutation of the second allele. Donehower et al., performed a comprehensive assessment of the P53 pathway involvement in 32 cancers reported by The Cancer Genome Atlas (TCGA) and found that in cancers with *TP53* mutations, there was a loss of the second allele due to mutation, chromosomal deletion, or copy-neutral loss of heterozygosity in 91% of the cases^14^. The current literature strongly supports the central role of *TP53* alterations in CLL^15^. Approximately 90% of CLL patients with del(17p) carry a *TP53* mutation and ~ 60% of patients with *TP53* mutations also harbour del(17p), as detected by FISH^16–19^. Even in the absence of a del(17p), *TP53* mutations appear to have an equally profound impact on outcome and are more frequent in patients with a poor prognosis and a higher genetic complexity^8,20^. Moreover, CLL sub-clones carrying *TP53* mutations can be positively selected upon treatment, ultimately becoming the prevalent expansion of an initially minor mutant component ^4,17,21,22^.

The mere presence of *TP53* mutations is not synonymous per se of the complete inactivation of the P53 pathway, since mutant P53 proteins are functionally and structurally heterogeneous^23^ and may impact on important clinical variables, in multiple, often subtle ways, as revealed by cell-based assays and animal models^24^. Moreover, while it is widely accepted that alterations of the *TP53* gene may influence the clinical course of CLL by causing resistance to chemotherapy, their impact on clonal expansion and disease progression is still not fully defined, especially in the earliest stages of the disease. With the aim of clarifying these points, we studied *TP53* alterations in patients enrolled in the O-CLL1 trial (clinicaltrial.gov identifier NCT00917540). In this observational protocol, Binet stage A CLL patients from several Italian Institutions were prospectively enrolled within 12 months of diagnosis (median: 2.3 months) and their evolution was followed. Because the aim of the protocol was to study the disease since the early stages, patients at more advanced stages (Binet stage B and C) were excluded from recruitment. A total of 469 patients from this well characterised cohort were investigated for *TP53* mutations using next generation sequencing (NGS) and for del(17p) using FISH. The possible influence of the *TP53* alterations on the time to first treatment (TTFT) and on the expansion of individual clones or sub-clones was also investigated.

## Results

### Incidence and characterization of the *TP53* mutational status in O-CLL1 patients

Binet stage A CLL patients from the O-CLL1 trial^25^, were characterised for cellular, molecular, and cytogenetic features at diagnosis (Supplementary Table 1A). *TP53* mutational status was determined by NGS on highly purified (> 95%) CLL B-cells from 475 patients. FISH analysis was used to determine the presence of del(17p) in 469 out of these 475 patients (Supplementary Table 1B). *TP53* alterations [del(17p) and/or *TP53* mutations] were identified in 31 patients. In five patients a *TP53* variant allele frequency (VAF) of approximately 50% was suggestive of a germline variant. A subsequent analysis of non-tumour samples revealed the presence of the same *TP53* mutation. Therefore, these were classified as *TP53* germ-line variants (Supplementary Table 1B). The remaining 26 patients were characterised by the presence of: (1) only a *TP53* mutation (group Mut/noDel = 15 cases), (2) a *TP53* mutation along with the deletion of del(17p) (group Mut/Del = 9 cases), and (3) del(17p) with no *TP53* mutation (group noMut/Del = 2 cases) (Table 1). The overall incidence of somatic *TP53* alterations in the O-CLL1 cohort [mutations: 5%; 24/469; del(17p): 2.3%; 11/469] and their distribution relative to del(17p) were similar to that of previous reports^4,16,17,21^. The VAF in the Mut/noDel group was below 50% and significantly lower (23.8 ± 18.4 vs 65.7 ± 35.7, *P* = 0.001) than that observed in the Mut/Del group. In addition, certain adverse prognostic factors, such as CD38 expression (*P* = 0.0006), unmutated-IGHV (UM-IGHV) gene status (*P* = 0.003), and *NOTCH1* mutations (*P* = 0.0415) were significantly less frequent in the cases with *TP53* mutation only (Table 1). Conversely, a favourable prognostic factor, such as del(13q), showed a trend toward a higher frequency only in Mut/noDel cases (*P* = 0.06) (Table 1).

**Table 1:** Biologic and molecular features of the 26 CLL patients harbouring somatic *TP53* alterations.

ID^#^	cDNA variant*	Protein variant*	VAF%^a^	del(17p)	del(13q)	+12	del(11q)	CD38	ZAP-70	IGHV^b^	*NOTCH1*	*SF3B1*
**15 patients with Mut/noDel**												
MS0273	c.524G>A	p.Arg175His	11.7	−	+	−	−	−	−	M	WT	WT
CA0082	c.638G>T	p.Arg213Leu	7.7	−	+	−	−	−	−	M	WT	WT
CF0003	c.833C>G	p.Pro278Arg	4.1	−	+	−	−	−	−	M	WT	WT
GM0252	c.584T>C	p.Ile195Thr	23.9	−	+	−	−	−	+	M	WT	WT
MG0248	c.844C>T	p.Arg282Trp	44.6	−	+	−	−	−	+	M	WT	WT
DD0478	c.568_570delCCT	p.Pro191del	18.3	−	+	−	−	−	+	M	WT	WT
GC0448	c.742C>T	p.Arg248Trp	2.8	−	+	−	+	−	−	M	WT	WT
PA0254	c.481G>A	p.Ala161Thr	38.0	−	+	−	−	−	+	M	WT	WT
AR0222	c.578A>G	p.His193Arg	41.5	−	+	−	−	−	−	M	WT	WT
CG0620	c.626_627delGA	p.Arg209fs	48.7	−	+	−	−	−	−	UM	WT	WT
AA0396	c.470T>A	p.Val157Asp	4.4	−	+	−	−	−	+	UM	WT	WT
CR0115	c.338T>C	p.Phe113Ser	2.0	−	−	−	−	−	−	M	WT	WT
AG0464	c.517G>A	p.Val173Met	48.5	−	+	−	−	−	−	M	WT	WT
SG0168	c.260_261dupC	p.Ala88fs	34.8	−	+	−	−	−	−	M	WT	WT
DA0455	c.818G>A	p.Arg273His	18.0	−	−	−	−	−	+	M	WT	WT
**9 patients with Mut/Del**												
CR0203	c.772G>T	p.Glu258Ter	97.7	+	−	+	−	+	+	UM	Mut^d^	WT
IF0044	c.842A>G	p.Asp281Gly	95.1	+	−	−	−	+	+	UM	WT	WT
DA0094	c.833C>G	p.Pro278Arg	98.3	+	+	−	−	+	+	UM	WT	WT
CS0290	c.814G>T	p.Val272Leu	36.6	+	−	−	−	−	−	M	WT	WT
DG0193	c.584T>C	p.Ile195Thr	77.3	+	+	−	−	−	+	UM	Mut^d^	WT
FD0404	c.824G>A	p.Cys275Tyr	63.6	+	−	−	−	+	+	UM	Mut^d^	WT
PG0028	c.524G>A	p.Arg175His	16.4	+	+	−	−	+	+	UM	WT	WT
CG0622	c.497C>A	p.Ser166Ter	11.0	+	+	−	−	+	+	M	WT	WT
DS0264	c.622_623ins^c^	p.Asp208Glufs	95.1	+	−	−	−	−	−	UM	WT	WT
**2 patients with noMut/Del**												
CP0036	WT	WT	n.a	+	−	−	−	+	−	M	WT	WT
NT0628	WT	WT	n.a	+	−	−	−	+	+	UM	WT	WT

^#^Patient identification; *Based on HGVS (Human Genome Variation Society) nomenclature; ^a^VAF, Variant allele frequency; ^b^IGHV-UM, unmutated; ^b^IGHV-M, mutated; ^c^insertion of AATTTGGATG; ^d^Mut, *NOTCH1* coding mutation c.7541_7542delCT, p.P2515fs*4; + 12, trisomy 12; WT, wild-type; n.a., not applicable. Significant P-values regarding the comparison of Mut/noDel *vs* Mut/Del patients for the presence of del(13q), CD38, UM-IGHV and *NOTCH1* mutation are the following: *P* = 0.06, *P* = 0.0006, *P* = 0.003 and *P* = 0.0415, respectively.

### Influence of the *TP53* status on the TTFT in O-CLL1 patients

**Figure 1:**
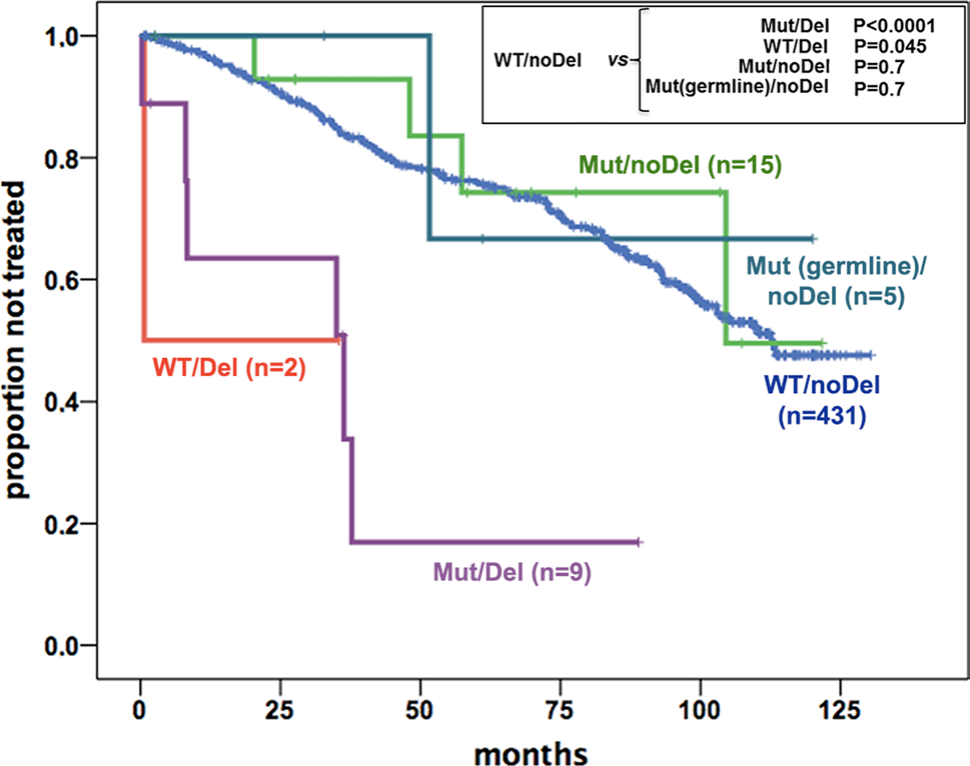
Analysis of the prognostic impact of *TP53* alterations in the O-CLL1 cohort. Kaplan–Meier curves of cases differently clustered on the basis of the presence of a *TP53* mutation and del(17p). O-CLL1 patients were divided in different groups: WT/noDel patients with neither a *TP53* mutation nor del(17p); Mut/noDel and Mut (germline)/noDel, patients with a *TP53* mutation (somatic or germline, respectively) and without del(17p); Mut/Del, patients that show both a *TP53* mutation and del(17p); WT/Del, patients harbouring only del(17p).

Next, the influence of the *TP53* status on TTFT was evaluated in the O-CLL1 cohort. After a median follow-up of 86 months, TTFT data were available for 462/469 patients, including the 5 cases with a *TP53* germline variant. The TTFT of patients with somatic *TP53* mutations (Mut = Mut/noDel + Mut/Del: 24 patients) was not statistically different from that of cases with wild-type *TP53* (WT/noDel: 431 patients) (HR = 1.5, 95% CI 0.8–2.7, *P* = 0.7). In contrast, the TTFT was significantly shorter when patients with del(17p) (Del = Mut/Del + noMut/Del: 11 patients) were compared to those without deletion (noDel: 446 patients, excluding the 5 with *TP53* germline variant) (HR = 5.2, 95% CI 2.4–11.2, *P* < 0.0001). A multivariate analysis, in which only the presence of del(17p) and of *TP53* mutations were introduced, indicated that the presence of the del(17p) at diagnosis constituted a significant risk factor for a shorter TTFT (HR 6.1, 95% CI 2.2–17.0, *P* = 0.001), independently of the presence of a *TP53* mutation (HR 0.8, 95% CI 0.4–1.8, *P* = 0.6). The cohort analysed was also stratified according to the presence/absence of *TP53* mutations and/or del(17p) in different combinations (see Fig. 1). The 9 Mut/Del and the 2 WT/Del patients had a significantly shorter TTFT than the 431 WT/noDel cases, whereas a somatic *TP53* mutation in the absence of del(17p) (Mut/noDel: 15 patients) was not predictive of a shorter TTFT. A similar result was obtained when the five cases with germ-line allelic variants were compared with the WT *TP53* cases. Subsequently we addressed the issue of whether the VAF in the Mut/noDel cases could have an influence on the prediction of the TTFT. To this end Mut/noDel cases were stratified according to high (> 10%) and low (< 10%) VAF values and the TTFT of the two groups was analysed. No significant differences were detected in the TTFT in the two groups of patients. The clinical impact of *TP53* mutations with different VAF also was analysed in the group of cases with mutated IGHV genes to eliminate a possible confounding factor represented by a strong predictor of prognosis such as IGHV gene mutation status. In this group, there were 9 patients with a VAF > 10% and 4 patients with a VAF < 10%. However, no statistically significant differences in the TTFT were observed (*P* = 0.5) (Supplementary Fig. 1). Therefore, the size of the subclone characterised by the presence of a *TP53* mutation did not appear to influence the TTFT in Binet stage A CLL. The presence of del(17p), although mostly associated with a *TP53* mutation, appears to be more deleterious than the presence of the a *TP53* mutation itself in determining a shorter TTFT.

### Functional studies on mutant P53 proteins from O-CLL1 patients

Next, the functional properties of the P53 proteins encoded by the *TP53* mutations detected in our cohort were investigated. A well-established yeast-based functional assay was employed, using four reporter strains. These strains have a different P53 Response Element (RE), which is derived from the promoter of one of four human P53 effector, i.e. P21, BAX, MDM2, and PUMA, respectively. The residual transactivation ability of the mutant P53 proteins compared to that of the WT protein (set as the 100% value) was measured at 30 °C and 37 °C (Table 2). With a threshold of residual WT P53 activity of 20%, all mutant P53 proteins (excluding those encoded by *TP53* germline variants) were found inactive at 37 °C, whereas some activity was observed at 30 °C (Table 2; Supplementary Fig. 2A). In fact, five mutants encoded P53 proteins with residual activity (p.Ala161Thr, p.Pro191del, p.Arg213Leu, and p.Arg282Trp within the Mut/noDel group and p.Val272Leu within the Mut/Del group). Of note, the P53 protein encoded by all *TP53* germline variants (p.Asn235Ser, p.Arg283Cys, and p.Gly360Val) behaved similarly to the WT P53 and were active at both 30 °C and 37 °C (Supplementary Table 3).

**Table 2:** Evaluation of the transactivation ability of somatic *TP53* mutations using a yeast-based assay.

ID	Protein variant	Mutant P53 residual activity (% of WT P53)							
		30 °C				37 °C			
		P21	PUMA	MDM2	BAX	P21	PUMA	MDM2	BAX
**15 patients with Mut/noDel**									
MS0273	p.Arg175His	0	1	1	1	0	1	1	1
CA0082	p.Arg213Leu	54	8	21	7	2	2	3	3
CF0003	p.Pro278Arg	0	1	1	1	1	3	3	3
GM0252	p.Ile195Thr	6	2	2	2	1	3	3	4
MG0248	p.Arg282Trp	70	67	50	52	1	2	3	3
DD0478	p.Pro191del	67	38	55	42	2	3	4	5
GC0448	p.Arg248Trp	0	1	1	1	1	2	3	3
PA0254	p.Ala161Thr	96	62	77	74	2	2	4	4
AR0222	p.His193Arg	0	1	1	1	1	1	3	3
CG0620	p.Arg209fs	0	1	1	1	1	1	2	3
AA0396	p.Val157Asp	0	1	1	1	1	2	3	3
CR0115	p.Phe113Ser	3	1	1	1	1	3	3	3
AG0464	p.Val173Met	0	1	1	1	1	3	3	2
SG0168	p.Ala88fs	1	1	1	1	2	4	5	7
DA0455	p.Arg273His	0	1	1	1	1	1	3	4
**9 patients with Mut/Del**									
CR0203	p.Glu258Ter	0	1	1	1	0	1	1	3
IF0044	p.Asp281Gly	0	1	1	1	1	3	3	3
DA0094	p.Pro278Arg	0	1	1	1	1	3	3	3
CS0290	p.Val272Leu	87	64	77	74	4	3	3	3
DG0193	p.Ile195Thr	6	2	2	2	1	3	3	4
FD0404	p.Cys275Tyr	0	1	1	1	1	3	3	3
PG0028	p.Arg175His	0	1	1	1	0	1	1	1
CG0622	p.Ser166Ter	1	1	1	1	1	3	3	3
DS0264	p.Asp208Glufs	0	1	1	1	1	3	3	3

Results are shown as residual activity of the mutant P53 protein with respect to wild-type (WT) P53 protein set as 100%. Each single mutant P53 was expressed in four different reporter yeast strains identified by the P53 Response Element (RE) from the promoter of the P21, PUMA, MDM2 or BAX effector genes. Transactivation ability was determined by growing yeast at 30 °C and 37 °C.

**Table 3:** Evaluation of the dominant-negative potential of somatic *TP53* mutations using a yeast-based assay.

ID	Protein variant	% Net activity (P21, 30 °C)	Classification
**15 patients with Mut/noDel**			
MS0273	p.Arg175His	61	D
CA0082	p.Arg213Leu	> 100	r
CF0003	p.Pro278Arg	73	D
GM0252	p.Ile195Thr	> 100	r
MG0248	p.Arg282Trp	> 100	r
DD0478	p.Pro191del	> 100	r
GC0448	p.Arg248Trp	39	D
PA0254	p.Ala161Thr	> 100	r
AR0222	p.His193Arg	59	D
CG0620	p.Arg209fs	> 100	r
AA0396	p.Val157Asp	61	D
CR0115	p.Phe113Ser	> 100	r
AG0464	p.Val173Met	40	D
SG0168	p.Ala88fs	> 100	r
DA0455	p.Arg273His	32	D

Results are shown as percentage of the activity of the co-expression of wild-type (WT) and mutant P53 proteins with respect to the expression of the single WT P53 set as 100%. P53 proteins (WT and mutant) were co-expressed in yLFM-P21-5′ reporter strain and grown at 30 °C. Mutant P53s are classified as dominant (D) or recessive (r), when the net activity is below or above 100%, respectively.

Mutant P53 proteins were also transiently expressed in HCT116 *TP53*^−/−^ cells and were tested for their ability to transactivate the luciferase reporter gene carrying a fragment of the P21 promoter (Supplementary Fig. 2B). The results obtained were consistent with those obtained in yeast at 30 °C, confirming that some mutants (e.g. p.AlaA161Thr, p.Pro191del and p.Arg282Trp) had a partial activity. Finally, the *TP53* germ-line allelic variants identified in our cohort were found to encode proteins with a high functional efficiency of transactivation (p.Asn235Ser, p.Arg283Cys, and p.Gly360Val) in both mammalian cell- and yeast-based assays (Supplementary Fig. 2B and Supplementary Table 3).

It is known that certain mutant P53 proteins have the capacity of inhibiting the activity of the WT protein when heterozygous at *TP53* locus [i.e. they are Dominant Negative (DN)]^26^. Therefore, the DN potential of each mutant P53 protein of the Mut/noDel group was determined using one of the reporter strains (i.e. yLFM-P21-5′) (Table 3). None of the mutant P53 proteins with partial residual function (i.e. p.Ala161Thr, p.Pro191del, p.Arg213Leu, p.Val272Leu and p.Arg282Trp) exhibited DN potential (Table 3). Of note, all of the *TP53* germline variants identified in patients without del(17p) encoded a recessive mutant P53 (Supplementary Table 4).

### Influence of the residual transactivation ability and DN potential of mutant P53 proteins on TTFT in O-CLL1 patients

**Figure 2:**
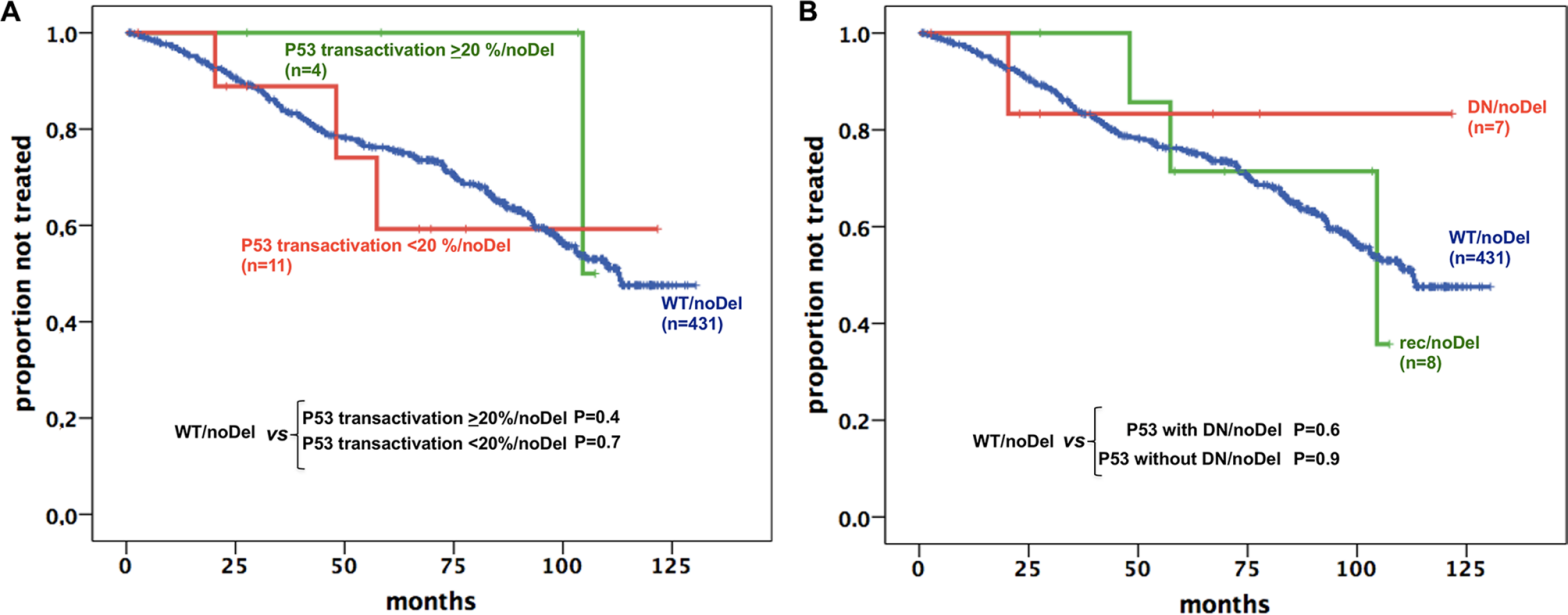
Influence of residual functionality of mutated P53 proteins on TTFT in O-CLL1 patients. Kaplan–Meier curves of cases belonging to the MUT/noDel group, clustered on the basis of (**A**) residual transactivation ability of the mutated P53 protein (≥ 20% versus < 20% with respect to WT P53) as determined in the yeast reporter strain yLFM-P21-5′; (**B**) dominant-negative (DN) or recessive (rec) classification of the mutated P53 protein, as determined in the yeast reporter strain yLFM-P21-5′.

Given the functional heterogeneity of P53 proteins in our cohort, we asked whether such heterogeneity could influence the TTFT. This analysis was carried out on the Mut/noDel patients’ group (15 cases). Neither the transactivation ability (Fig. 2A) nor the DN potential (Fig. 2B) appeared to affect the Kaplan–Meier curves of these patients’ groups, which were similar to those of the WT/noDel patients (431 patients). Although potentially interesting, these tests could not be carried out in the Mut/Del patients’ group since the residual transactivation activity was < 20% in 8/9 available cases and ≥ 20% in a single case. Thus, this issue warrants re-evaluation in a larger study sample.

### Multivariate analysis

**Figure 3:**
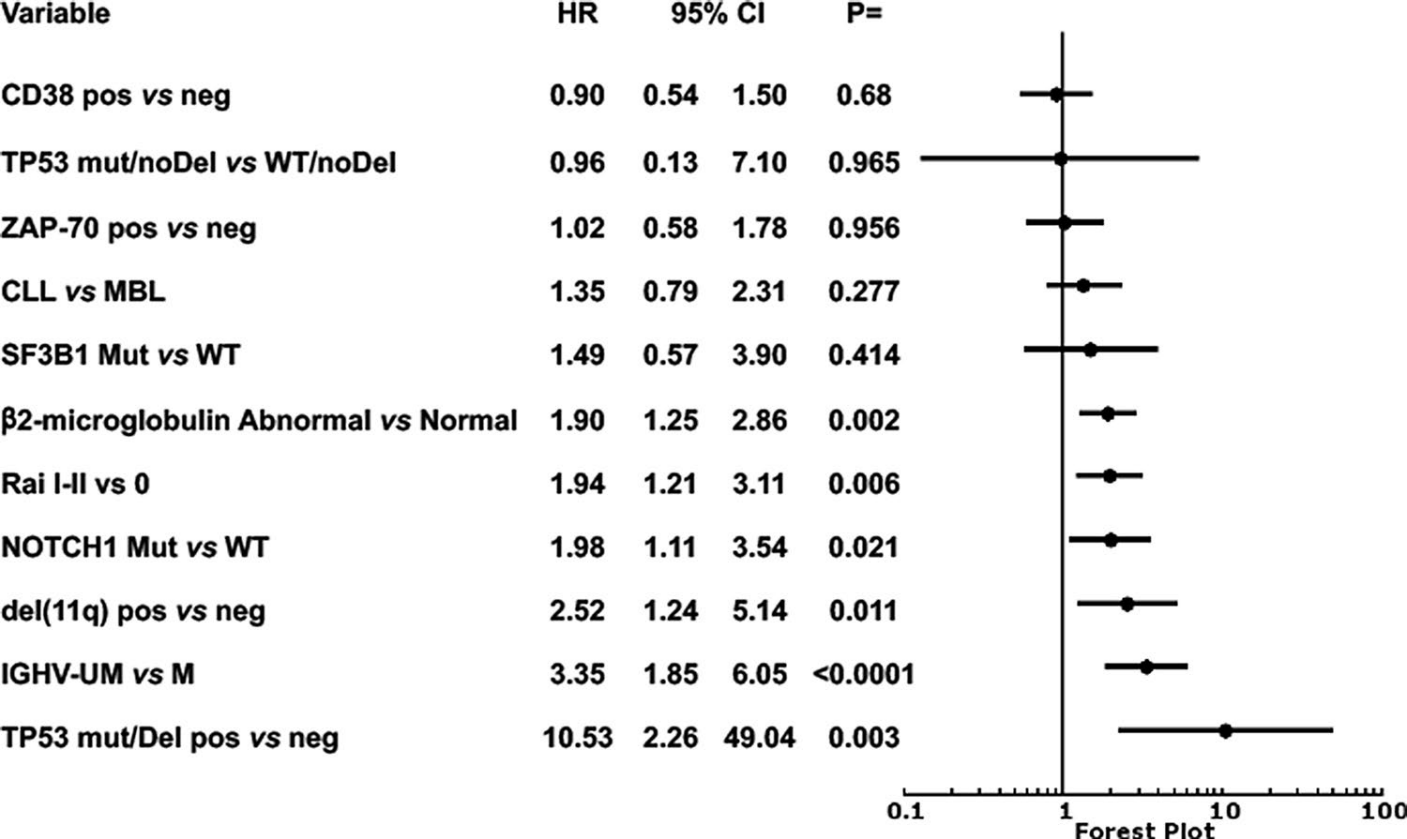
Analysis of the prognostic impact of *TP53* alterations in O-CLL1 cohort study. Cox multivariate analysis of biological and clinical variables found to be significant on univariate analysis. Presence of a *TP53* mutation associated with del(17p) was forced in the model as measure of P53 dysfunction.

CD38 and ZAP-70 expression, β2-microglobulin (β2M) serum levels, Rai stage, IGHV mutational status, del(11q), *NOTCH1* and *SF3B1* mutations, MBL classification were confirmed to have a significant prognostic power on univariate analysis^27^ (Supplementary Table 5). A Cox multivariate analysis was then performed by introducing into the model markers indicative of *TP53* alterations together with the variables with a significant prognostic power for TTFT determined in the univariate analysis. Both del(17p) together with *TP53* mutations, and del(17p) alone, retained an independent prognostic power, whereas *TP53* mutation alone did not. Other markers with an independent prognostic power were IGHV mutational status, del(11q), *NOTCH1* mutation, β2M levels, and Rai stage (Fig. 3).

### Changes in the *TP53* alterations pattern with time

**Figure 4:**
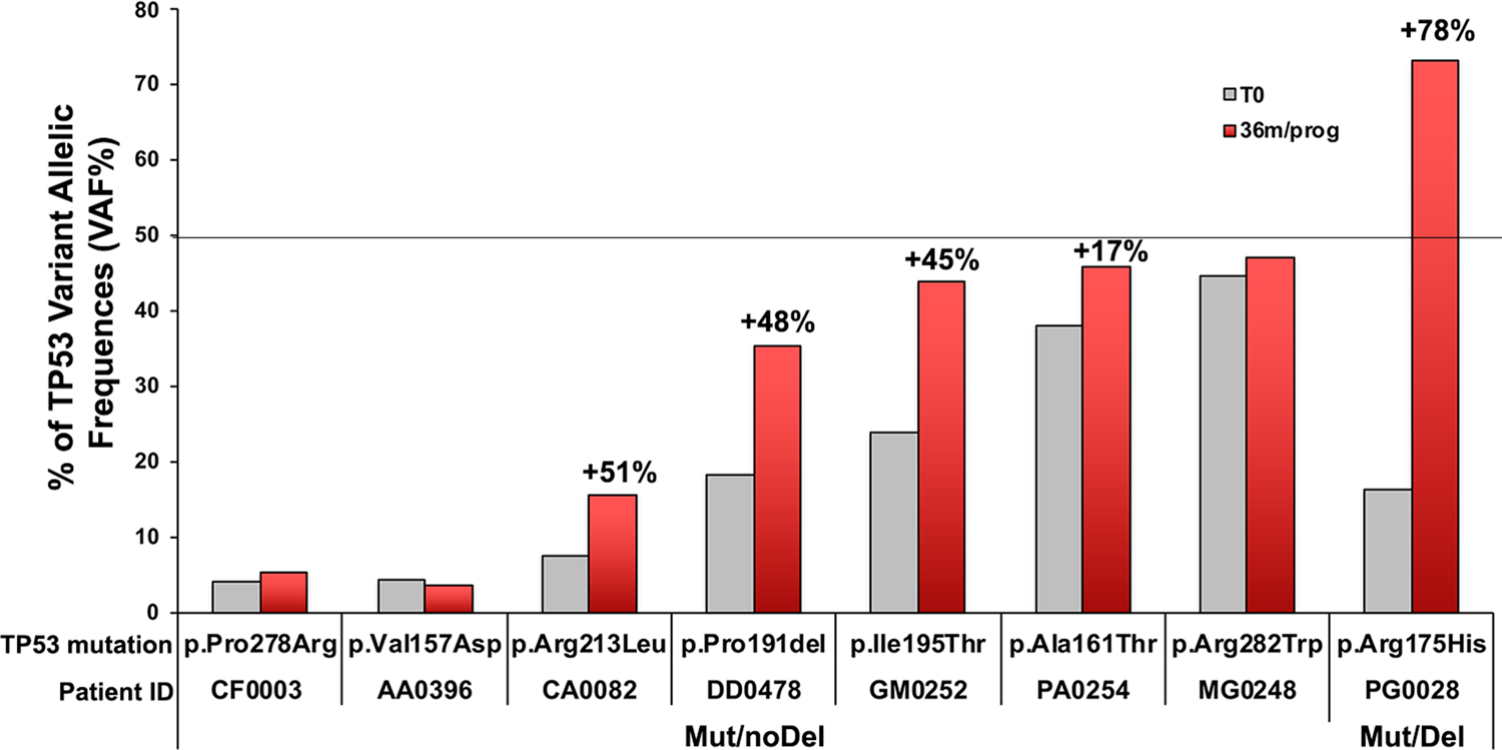
Determination of variant allele frequency (VAF) at 36 months after diagnosis or at progression in O-CLL1 cohort study. *TP53* mutational status was re-assessed in 171 patients by NGS after 36 months from the initial test or at disease progression. Presented are the 8 cases with a *TP53* mutation at diagnosis, which continued to express the same mutation, albeit with some degree of clonal expansion. FISH analysis for del(17p) was repeated in all cases after 36 months (36 m, red bars) or at the time of therapy need (prog, red bars): none of the cases negative for del(17p) (Mut/noDel) became del(17p) positive. Percentage increase in the VAF of the samples analysed at 36 m/prog, compared to the samples at T0 (grey bars), are indicated in the figure only for cases in which it clearly occurred (CA0082, DD0478, GM0252, PA0254, PG0028). The increment was calculated according to the formula: (VAF 36 m/prog–VAF T0)/VAF 36 m/prog × 100. Of the two cases that experienced progression, PG0028 presented an increase of the percentage of del(17p) positive cells and of VAF (78%). The other case (AA0396) showed no increase in VAF.

*TP53* mutational status was re-assessed in 171 patients after 36 months from diagnosis or at the time of therapy need. Of these, 140 re-tested at 36 months after diagnosis, did not present any new *TP53* alteration. Another 23 cases, without *TP53* alterations at diagnosis, were retested at the time of therapy need; again, no new *TP53* alterations were recorded. Eight cases with a *TP53* mutation at diagnosis were re-tested at 36 months (6 cases) or at the time of therapy need (2 cases). All of the 8 cases with a somatic *TP53* mutation continued to express the same mutation seen at diagnosis, although with some changes in their VAF (Fig. 4). The 163 patients, negative for somatic *TP53* mutations at diagnosis, still presented WT *TP53* gene (or the same germline *TP53* allelic variant) after 36 months. None of these 161 patients harboured del(17p) at diagnosis or at re-testing. The two cases re-tested at the time of therapy need presented p.Arg175His or p.Val157Asp, respectively. Of these, one [with p.Arg175His, and a del(17p) both at diagnosis and at re-testing] exhibited an over fourfold VAF increase, while the other [with p.Val157Asp mutation and no del(17p) also at re-testing] did not manifest any change in VAF.

## Discussion

The present study investigated the influence on disease progression of *TP53* alterations in Binet A CLL patients, enrolled in the multicentre prospective observational O-CLL1 trial with a long follow-up (median ~ 7 years). This influence was measured by determining the TTFT, which represents a rather accurate approach to assess disease progression. *TP53* mutations and del(17p) were determined in 98.7% of patients (469/475). There are differences in the thresholds distinguishing positive from negative cases for the FISH and NGS analysis (i.e. measurement of del(17p) and *TP53* mutations, respectively) due to the different sensitivity of each methodology. Since the sensitivity is somewhat imbalanced in favour of NGS, it cannot be excluded that certain patients harbour a del(17p), in addition to the *TP53* mutation, but that the former lesion goes undetected by FISH being present at sub-threshold level in a small sub-clonal component. Therefore, all the categories of *TP53* alterations dealt within the present paper refer to those detected within these technical limits. This admittedly may represent a limitation, especially in cases with VAF < 10%.

The overall incidence of somatic *TP53* mutations [5% (24/469 patients)] and del(17p) [2.3% (11/469 patients)] in the O-CLL1 cohort and their relative distribution are similar to that of previous reports^4,16,17,21^. The majority of *TP53* mutations were point mutations, causing a single amino-acid substitution in the P53 protein DNA binding domain. This was true for both the Mut/noDel and the Mut/Del patient groups. Although over 2,000 amino acid substitutions caused by a point mutation in the *TP53* gene have been identified, 3 amino acid substitutions (p.Arg175His, p.Pro278Arg, and p.Ile195Thr) were shared by the Mut/noDel and Mut/Del patients’ groups.

The biological features of Mut/Del and Mut/noDel CLL patients were significantly different, since adverse prognostic factors, such as CD38 positivity (*P* = 0.006), UM-IGHV gene status (*P* = 0.003), and *NOTCH1* mutations (*P* = 0.0415), were less frequently observed in the Mut/noDel patients’ group. We also observed that the TTFT of patients with mutated or WT *TP53* gene was similar (*P* = 0.7), a finding which is in line with that of Brieghel et al.^28^, who demonstrated that *TP53* mutations, determined by NGS, did not influence the clinical course of patients, in the absence of del(17p); this was true for cases with high (VAF ≥ 10%) and low (VAF < 10%) *TP53* mutation burden^28^. We also did not find differences in the TTFT of Mut/noDel patients when they were stratified into two groups based upon high or low VAF, and this was also true for the patients with *TP53* mutations from the mutated IGHV group stratified according to VAF values. Therefore, the size of the cell sub-clone bearing the *TP53* mutations does not appear to influence disease progression for Binet stage A CLL patients. In this study, the TTFT of the patients with del(17p) was significantly shorter than that of patients without deletion (*P* < 0.0001), while del(17p) concomitant with a *TP53* mutation represented, an additional and independent prognostic factor associated with shorter TTFT in multivariate analyses. However, in interpreting these data, it should be noted that most patients with del(17p) also had a *TP53* mutation, and that only two cases had del(17p) without a *TP53* mutation. Therefore, it is difficult to precisely evaluate the contribution of del (17p) alone to disease progression with this low number of cases. However, it is of note that Yu et al*.*^29^ reported that Mut/Del CLL patients had a TTFT shorter than that of the Mut/noDel patients. Moreover, Hoechstetter et al.^30^ showed that del(17p) is the highest weighted factor of the six considered in a multivariate analysis to predict TTFT and OS in Binet stage A CLL, although it requires the cooperation of additional factors to determine progression.

Unlike that reported in this and in the other studies quoted above, Dicker et al.^18^ and Rossi et al.^5^ reported that *TP53* mutations alone were capable of predicting a shorter TTFT. These discrepancies are difficult to explain also given the differences in methodologies used, clinical study design, and cohorts investigated. However, it is worth underlining that a predominance of Binet B and C stage patients was included in those studies, whereas we focused on Binet A cases only.

The mutant P53 proteins encoded by *TP53* mutations detected in this study were investigated further for their transactivation ability and DN potential. Although mutant P53 proteins appeared to be functionally heterogeneous, such heterogeneity was not associated with differences in TTFT within the Mut/noDel patients’ group. However, the interpretation of these data requires caution because of the relatively low number of Mut/noDel cases and further analyses in larger cohorts seem to be needed. Furthermore, it is worth recalling that mutant P53 proteins can acquire new functions favouring tumour cell expansion. These properties, indicated collectively as gain of function^24^, may in principle be present in CLL patients and perhaps have a higher incidence in more advanced cases. These too deserve analyses in large cohorts of patients.

IGHV mutational status represents a valuable prognostic marker for risk progression and outcome^11^. In the O-CLL1 cohort, the majority of cases with del(17p) had unmutated IGHV genes, whereas the majority of Mut/noDel cases had mutated IGHV genes. This finding may concur to explain why Mut/noDel cases progressed more slowly to the more advanced stages requiring therapy (Table 1). Furthermore, Mut/noDel cases were mostly negative for other unfavourable prognostic markers, whereas this was not the case for the group of patients with del(17p) (Mut/Del and WT/Del) (Table 1). These considerations may also help explain the discrepancies in the prognostic role of *TP53* mutations between this study and those including Binet B and C patients. Since unmutated-IGHV cases are likely to progress more rapidly towards advanced stages^31,32^, it is possible that cases with mutated IGHV genes and *TP53* mutations, that may not progress, are less frequently found in cohorts comprising numerous advanced cases.

Re-evaluation of 171 patients from this study following a 36 months interval or at the time of therapy need did not reveal any changes in *TP53* alterations status. Specifically, there was neither an acquisition/loss of a *TP53* mutation nor of del(17p). Only in one patient an expansion of a sub-clone carrying a non-functional *TP53* mutation was observed at progression.

In conclusion, the present study based on a clinically and molecularly well-characterised Binet stage A CLL cohort demonstrates that the occurrence of del(17p) significantly predicted TTFT, while that of a *TP53* mutation alone, was unable of such prediction.

## Materials and methods

All methods were carried out in accordance with relevant guidelines and regulations.

### Patients and CLL cells preparation

Only previously untreated Binet stage A CLL patients not requiring therapy according to NCI guidelines were prospectively enrolled within 12 months of diagnosis (O-CLL1 protocol, clinicaltrial.gov identifier NCT00917540). This protocol was presented by the Gruppo Italiano Studio dei Linfomi (GISL) on behalf of several Italian participating institutions and approved by the Ethics Review Committee (Comitato Etico Provinciale, Modena, Italy). Written informed consent was obtained from all patients in accordance with the declaration of Helsinki. The ethics committees from each participating centre (listed in the acknowledgements) approved this study. The median time between diagnosis and patient enrolment in the study was 2.3 months. A total of 420 (89.6%) and 386 (82.3%) patients had follow-up data at 1- and 2-year, respectively. Recruitment began in January 2007 and the criteria for CLL diagnosis employed followed the 1996 NCI/WG guidelines requiring > 5000 lymphocytes/µL in the peripheral blood. One hundred thirty-six cases (26%) fulfilled the definition of MBL (i.e. < 5.0 × 10^9^ B lymphocytes/L in the peripheral blood and no apparent lymph node, spleen, or liver enlargement) according to the more recent NCI/IWCLL classification^33^. Treatment was decided uniformly for all participating centres based on documented progressive and symptomatic disease according to National Cancer Institute-sponsored working guidelines^33^. CLL cell phenotypes, CD38, and ZAP-70 expression, and IGHV mutational status assessment was centralised in the laboratory in Genoa, while all FISH assays were performed in Milan.

The median age of the entire cohort was 61.2 years, 214 cases (41%) were female. Finally, at the time of the present analysis, 179 cases (35.2%) progressed and were treated.

### Ethical parameters

Ethical parameters, included in the synopsis of O-CLL1 protocol, clinicaltrial.gov identifier NCT00917540 were the following: It is responsibility of the investigator(s) to submit a copy of the protocol and detailed patient information sheet-consent form to an Independent Ethics Committee or Institutional Review Board in order to obtain independent approval to conduct the study. It is responsibility of the investigator(s) to ensure that the study is conducted in full conformance with the principles of the current version of the Declaration of Helsinki and to ensure that the study is performed in accordance with the international Good Clinical Practice (GCP) standards and according to all local laws and regulations concerning clinical studies).

### CLL cells preparations

Peripheral blood mononuclear cells (PBMCs) from patients with CLL were isolated by Ficoll-Hypaque (Seromed, Biochrom) density gradient centrifugation. CD19-positive CLL cells were enriched by negative selection with the EasySep-Human B-cell Enrichment Kit without CD43 depletion (STEMCELL Technologies, Voden Medical Instruments S.p.A.), using the fully automated protocol of immunomagnetic cell separation with RoboSep (Stem Cell Technologies). The percentage of purified B Cells (CD19+) exceeded 95%, as detected by flow cytometry^34^.

### CD38 and ZAP-70 determination, FISH analyses, *NOTCH1* and *SF3B1* mutations, and IGHV gene analysis

Heparinized blood samples were obtained and immediately shipped to the Genoa laboratory at room temperature, to arrive on the same or the following day; the cells were immediately processed. In these conditions, repeated quality control tests indicated minimal cell apoptosis or necrosis as measured by flow cytometric analysis using annexin V or propidium iodide (PI) staining. CD38 positive leukemic cells were measured by triple staining with CD19 fluorescein isothicyanate (FITC), CD38 phycoerythrin (PE), and CD5 Cy-Chrome (Becton Dickinson & Co., Sunnyvale, CA, USA). The cells were analysed using a FACS Calibur flow cytometer (Becton Dickinson & Co.) as previously described^35^. ZAP-70 was determined by flow-cytometry. CLL cells were first incubated with CD3 PE-CY7, CD19 PE and CD5 allophycocyanin (APC) monoclonal antibodies (mAbs) (Becton Dickinson & Co.), fixed, permeabilised with Fix and Perm reagents (Caltag Laboratories) and exposed to a ZAP-70 FITC (Upstate, Lake Placid, NY, USA) or an isotype control mAb (mouse IgG2a FITC; Becton Dickinson). Cytogenetic abnormalities involving deletions at chromosomal loci 11q22.3, 13q14.3, 17p13.1, and trisomy 12 were evaluated by FISH in a purified CD19+ population, as previously described^36^. The FISH study was performed using the protocol provided by the manufacturer of the multicolour probes LSI D13S25/LSI 13q34, LSIp53/CEP17, LSI ATM/CEP11, and CEP12 (Abbot Park, IL, USA). A total of 200 interphase nuclei were analysed for each probe set. The cut-off points (mean + 3 standard deviations) for positive values assessed on peripheral mononuclear cells from ten control subjects were 3.4%, 1.7%, 3.8% and 3.4% for + 12, del(11q), del(13q), and del(17p), respectively^36^.

IGHV mutational status was assessed using cDNA, as previously described^37^. Sequences were aligned to the IMGT directory and analysed using IMGT/VQUEST software. *SF3B1* (exons 14, 15, and 16, including splice sites; RefSeq NM_012433.2) genes were analysed by PCR amplification and Sanger sequencing of high molecular weight genomic DNA extracted from CD19+ purified B-cells (> 95%) as previously described^38^. The *NOTCH1* c.7541_7542delCT mutation was tested by NGS using Roche 454 technology and subsequently validated by Amplification Refractory Mutation System (ARMS)-PCR as previously described^39^.

### Evaluation of *TP53* mutations

The *TP53* mutational status of all cases (n = 475) was analysed by NGS using Roche Junior (Roche-454 Life Sciences, Penzberg, Germany) in 250 cases as previously described^40^ and by the Ion Torrent platforms (Thermo Fisher Scientific Carlsbad, CA) in 241 cases (see https://tools.thermofisher.com/content/sfs/manuals/MAN0013432_Ion_AmpliSeq_Library_Prep_on_Ion_Chef_UG.pdf for further details)*.* Sixteen cases were tested using both platforms with 100% concordance. Briefly, *TP53* libraries were prepared using genomic DNA extracted from CD19+ purified B cells (QIAamp DNA Blood Mini Kit, Qiagen Hilden, Germany) according to the respective protocols (see above) and then sequenced with both NGS platforms taking into account the achievement of a threshold of 500 reads for each *TP53* amplicon. Cases with VAF ≥ 2% were considered positive for *TP53* mutation; in addition, samples with *TP53* variant < 10% VAF were re-evaluated by a second NGS run. The panel utilized for the Ion torrent NGS study was the Ion AmpliSeq™ *TP53* Panel comprising 24 primer pairs across 2 pools that provides 100% of coverage of exons 2–11 and exon–intron boundaries (± 30 bp padding). The *TP53* primer panel applied to Roche NGS technology spanned exons 4 to 9^40^. Of the 171 cases sequenced (131 with Roche J, 51 with Ion Torrent, 11 were subsequently analysed using both sequencing approaches with concordant results), *TP53* status was determined not only at the time of diagnosis, but also after 36 months or at the time of therapy need. Any *TP53* variant identified [exonic, intronic and frequent SNPs (e.g. Pro72Arg)] identified by NGS, was recorded for each sample. For all samples in which the variant was verified, and the VAF was approximately 50%, suggesting a germline origin, the patient-matched normal tissue DNA was subsequently analysed by NGS. For patients CG0015 and RA0023, DNA was obtained from buccal swabs (3 separate swabs per patient). For GS0473, RC0479, and NF0056 non-tumour DNA was obtained from CD3+ cells obtained by triple staining with CD19, CD5, and CD3 mAbs (Becton Dickinson) followed by cell sorting (FACS ARIA II, Becton Dickinson) of CD3+ CD5+ cells to avoid any contamination of CD19+ CD5+ neoplastic cells^10^. No mutations in exons 2, 3, or 11 were identified by Ion Torrent NGS among the 241 CLL cases analysed using this platform, while in only one case (1/241, 0.4%) a germline variant, was discovered in exon 10. This suggests that there was a number of mutated samples, possibly not being detected by sequencing of exons 4–9 with the Roche platform, that was negligible or non-existing, and that the results obtained with the two methods were comparable. Overall, sequencing of the entire coding portion of the gene using the Ion AmpliSeq™ *TP53* Panel indicated that exons 4–8 were the most recurrently mutated. The functional analysis, performed using a yeast-based assay, was applied, to those *TP53* variants causing either amino-acid substitutions or coding for a truncated P53 protein.

### Evaluation of P53 function in a yeast-reporter assay

#### Yeast strains and media

The yLFM-P21-5′, yLFM-PUMA, yLFM-MDM2P2C, and yLFM-BAX A + B yeast strains were used to assess the functionality of *TP53* variants: all strains were isogenic except for the different P53 response element (RE) located upstream of the luciferase reporter gene (LUC1). Cells were grown in YPDA medium (1% yeast extract, 2% peptone, 2% dextrose, 200 mg/L adenine) or in selective medium (with or without 2% agar) containing dextrose or raffinose as a carbon source plus adenine (200 mg/L), but in the absence of tryptophan and/or leucine (Sigma-Aldrich, Saint Louis, Missouri, USA; Biokar Diagnostics, Allonne, France). Galactose (Sigma-Aldrich, Saint Louis, Missouri, USA) was added to the medium to modulate P53 expression under the inducible GAL1,10 promoter.

#### Yeast vectors

For the transactivation assay, human WT and mutant P53 proteins were expressed using a pTSG-based vector (TRP1). The P53 mutants were constructed in the pTSG-based vector (through SgraI/StuI digestion and subsequent ligation) (New England Biolabs) from available pLS-based vector^26,41^. When cloned mutants were not available, P53 mutants were firstly constructed in a pLS- or pTS-based vector exploiting in vivo yeast homologous recombination, as previously described^42^ and then cloned (through SgraI/StuI or XhoI/NotI digestion and subsequent ligation) (New England Biolabs) in the pTSG-based vector.

For the dominance assay, P53 mutant protein was expressed using a pLS- or pTS-based vector; the pLS89 (TRP1) or pLLS89 (LEU2) expressing WT P53 protein under the inducible GAL1,10 promoter was co-transformed in yeast, based on the previously used selection marker. Plasmid pRS314 (TRP1) and pRS315 (LEU2) were used as empty vectors. The list of primers used to construct pLS- or pTS-based P53 mutant vectors is available upon request.

#### Yeast functional assay

Quantitative functional assays (evaluation of transactivation ability and DN potential) were performed according to the miniaturized protocol we developed^43^. The transactivation activity of a mutant P53 was measured by calculating the percentage with respect to WT P53 (set as 100%); the DN potential was calculated by comparing the net activity of WT and mutant P53 co-expression with respect to the expression of WT P53 alone (set as 100%). A mutant P53 was defined as recessive or dominant when the net activity was above or below 100%, respectively.

### Evaluation of P53 function in a mammalian reporter-assay

#### Cell line and media

HCT116 *TP53*^−/−^ cells (human colon carcinoma) were obtained from Dr. B. Vogelstein (The Johns Hopkins Kimmel Cancer Center, Baltimore, MD). Cells were grown in RPMI containing 10% foetal bovine serum, L-glutamine, and a penicillin–streptomycin antibiotic mixture (Euroclone, Milano, Italy), and maintained at 37 °C in 5% CO2 at 100% humidity.

#### Mammalian expression and reporter vectors

A pCIneo-based (Promega) vector was used to express WT and mutant P53 in mammalian cells. Plasmids expressing mutant P53 were constructed as previously described^42^. The pGL3-1138 and the pRL-SV40 plasmids were used as reporter (P21 promoter) and normalization vectors, respectively^44^.

#### Mammalian functional assay

HCT116 *TP53*^−/−^ cells transfected with pCI-neo-based P53 expression vectors, the reporter, and normalization plasmids, were collected and washed with cold PBS. Lysis was performed in 1X PLB buffer (Passive Lysis Buffer, Promega). Luciferase assays were conducted as previously described^45^.

### Statistical analysis

Statistical analyses were performed as previously described^25,27^. Briefly, SPSS for Windows, v13.0, 2004 software (SPSS, UK) was used for all the analyses. We performed, statistical comparisons, for categorical variables, using two-way tables for the Fisher's exact test, while multiway tables were used for the Pearson's Chi-square test. TTFT analyses were performed using the Kaplan–Meier method in patients with a minimum follow-up. Using the log‐rank test, we calculated the statistical significance of associations between individual variables and survival. We investigated the prognostic impact for the outcome variable by univariate and multiple Cox regression analysis. Data were expressed as hazard ratio (HR) and 95% confidence interval (CI). A value of *P* < 0.05 was considered statistically significant.

## Supplementary information

Supplementary information.
