# Supplementary material for: Time to first treatment and P53 dysfunction in chronic lymphocytic leukaemia: results of the O-CLL1 study in early stage patients
Source: Sci Rep. 2020 Oct 28;10:18427. doi: 10.1038/s41598-020-75364-3 (PMC7595214; doi:10.1038/s41598-020-75364-3)
Supplement: Supplementary file 1 — Supplementary information. [file 41598_2020_75364_MOESM1_ESM.doc]

Supplementary materials

Time to first treatment and P53 dysfunction in chronic lymphocytic leukaemia: Results of the O-CLL1 study in early stage patients

Paola Monti^1^, Marta Lionetti^2^, Giuseppa De Luca^3^, Paola Menichini^1^, Anna Grazia Recchia^4^, Serena Matis^5^, Monica Colombo^5^, Sonia Fabris^6^, Andrea Speciale^1^, Marzia Barbieri^6^, Massimo Gentile^7^, Simonetta Zupo^3^, Mariella Dono^3^, Adalberto Ibatici^8^, Antonino Neri^2,6^, Manlio Ferrarini^9^, Franco Fais^5,9^, Gilberto Fronza^1^*, Giovanna Cutrona^5^*, and Fortunato Morabito^4,10^*

**Affiliations**:

^1^Mutagenesis and Cancer Prevention Unit, IRCCS Ospedale Policlinico San Martino, 16132, Genoa Italy;

^2^Department of Oncology and Hemato-Oncology, University of Milan, 20122, Milan, Italy;

^3^Molecular Diagnostic Unit, IRCCS Ospedale Policlinico San Martino, 16132 Genoa, Italy;

^4^Biotechnology Research Unit, Aprigliano, A.O./ASP of Cosenza, 87100, Cosenza, Italy;

^5^Molecular Pathology Unit, IRCCS Ospedale Policlinico San Martino, Genoa, Italy;

^6^Hematology Unit, Fondazione IRCCS Ca’ Granda, Ospedale Maggiore Policlinico, 20122, Milan, Italy;

^7^Hematology Unit, Department of Onco-Hematology A.O. of Cosenza, 87100, Cosenza, Italy;

^8^Hematology Unit and Bone marrow transplantation, IRCCS Ospedale Policlinico San Martino, Genoa, Italy;

^9^Department of Experimental Medicine, University of Genoa, 16132, Genoa, Italy;

^10^Department of Hematology and Bone Marrow Transplant Unit, Augusta Victoria Hospital, Jerusalem, Israel.

*These authors contributed equally to this work

| **Supplementary Table 1A. Main biological features of O-CLL1 patients.** | | |
| --- | --- | --- |
| **Characteristic** | **Number of cases** | **%** |
| **CD38** | Total: 507 |  |
| Negative | 403 | 79.5 |
| Positive | 104 | 20.5 |
| **ZAP-70** | Total: 504 |  |
| Negative | 297 | 58.9 |
| Positive | 207 | 41.1 |
| **IGHV** | Total: 507 |  |
| Mutated | 354 | 69.8 |
| Unmutated | 153 | 30.2 |
| **FISH analysis** | Total: 499 |  |
| Negative | 177 | 35.4 |
| del(13q) | 224 | 44.8 |
| +12 | 55 | 11.0 |
| del(11q) | 32 | 6.4 |
| del(17p) | 11 | 2.2 |
| ***TP53* analysis** | Total: 475 |  |
| WT | 446 | 93.9 |
| Mutated | 29 | 6.1 |
| ***NOTCH1* analysis** | Total: 510 |  |
| WT | 445 | 87.3 |
| Mutated | 65 | 12.7 |
| ***SF3B1* analysis** | Total: 412 |  |
| WT | 396 | 96.1 |
| Mutated | 16 | 3.9 |
| **β2-microglobulin** | Total: 343 |  |
| Normal | 216 | 63 |
| Abnormal | 127 | 37 |

WT, wild-type

**Supplementary Table 1B. *TP53* status in 469 O-CLL1 patients analysed for the presence of both *TP53* mutation(s) and del(17p).**

**Characteristic Number of cases**

Patients with a somatic *TP53* mutation and no del(17p) 15

**Mut/noDel group**

Patients with a germinal *TP53* variant and no del(17p) 5

**Mut (germinal)/noDel group**

Patients with a somatic *TP53* mutation and with del(17p) 9

**Mut/Del group**

Patients with wild-type *TP53* and with del(17p) 2

**WT/Del group**

Patients with wild-type *TP53* and no del(17p)* 438

**WT/noDel group**

*Seven patients from this group were excluded from time to first treatment (TTFT) analysis due to the lack of follow-up data. TTFT analyses were therefore performed on data from 431 patients.

**Supplementary Table 2. Biologic and molecular features of the 5 CLL patients harbouring germinal *TP53* variants.**

_____________________________________________________________________________________________________________

**ID^#^ cDNA variant* Protein variant* VAF%^a^ del(17p) del(13q) +12 del(11q) CD38 ZAP-70 IGHV^b^ *NOTCH1*  *SF3B1***

_____________________________________________________________________________________________________________

GC0015 c.704A>G p.Asn235Ser 53.5 - - + - - + UM WT WT

RA0023 c.847C>T p.Arg283Cys 55.9 - - + - + + UM Mut^c^ Mut^d^

GS0473 c.847C>T p.Arg283Cys 50.0 - + - - - - M WT WT

RC0479 c.847C>T p.Arg283Cys 50.0 - + - - - - M WT WT

NF0056 c.1079G>T p.Gly360Val 53.7 - + - - - + M WT WT _____________________________________________________________________________________________________________

^#^Patient identification; *Based on HGVS (Human Genome Variation Society) nomenclature; ^a^VAF, Variant Allele Frequency; ^b^IGHV-UM, unmutated; ^b^IGHV-M, mutated; ^c^Mut, *NOTCH1* coding mutation c.7541_7542delCT, p.P2515fs*4; ^d^Mut, *SF3B1* coding mutation c.2221A>C, p.Lys741Gln; +12, trisomy 12; WT, wild-type.

**Supplementary Table 3.** **Evaluation of the transactivation ability of germinal *TP53* variants using a yeast-based assay**. Results are shown as residual activity of the mutant P53 protein with respect to wild-type (WT) P53 protein set as 100%. Each single mutant P53 was expressed in four different reporter yeast strains identified by the P53 Response Element (RE) from the promoter of the *P21*, *PUMA*, *MDM2* or *BAX* genes. Transactivation ability was determined by growing yeast at 30°C and 37°C.

**Mutant P53 residual activity (% of WT)**

**30°C 37°C** ___

**ID Protein variant P21 PUMA MDM2 BAX P21 PUMA MDM2 BAX**

GC0015 p.Asn235Ser 85 78 89 65 98 89 87 79

RA0023 p.Arg283Cys 93 33 27 52 76 33 13 30

NF0056 p.Gly360Val 89 97 100 100 82 91 84 91

GS0473 p.Arg283Cys 93 33 27 52 76 33 13 30

RC0479 p.Arg283Cys 93 33 27 52 76 33 13 30

**Supplementary Table 4.** **Evaluation of the dominant-negative potential of germinal *TP53* variants using a yeast-based assay.** Results are shown as percentage of the activity of the co-expression of wild-type (WT) and mutant P53 proteins with respect to the expression of the single WT P53 set as 100%. P53 proteins (WT and mutant) were co-expressed in yLFM-P21-5’ reporter strain and grown at 30°C. Mutant P53s are classified as dominant (D) or recessive (r), when the net activity is below or above 100%, respectively.

**ID Protein variant % Net Activity Classification**

**(P21, 30°C)**

GC0015 p.Asn235Ser >100 r

RA0023 p.Arg283Cys >100 r

NF0056 p.Gly360Val >100 r

GS0473 p.Arg283Cys >100 r

RC0479 p.Arg283Cys >100 r

**Supplementary Table 5. Univariate Cox regression analysis.**

**__________________________________________________________________________**

**Variable HR 95% CI P**

__________________________________________________________________________

CD38 (pos vs neg) 3.2 2.3-4.3 <0.0001

ZAP-70 (pos vs neg) 2.8 3.1-3.8 <0.0001

β2-microglobulin (abnormal vs normal) 2.2 1.5-3.1 <0.0001

Rai stage (0 vs I-II) 1.9 1.4-2.6 <0.0001

IGHV (UM vs M) 5.3 3.9-7.1 <0.0001

del(11q) (positive vs negative) 5.4 3.5-8.0 <0.0001

*NOTCH1* (Mut vs WT) 2.4 1.7-3.5 <0.0001

*SF3B1* (Mut vs WT) 2.4 1.2-4.8 <0.01

CLL vs MBL 2.3 1.5-3.4 <0.0001

__________________________________________________________________________


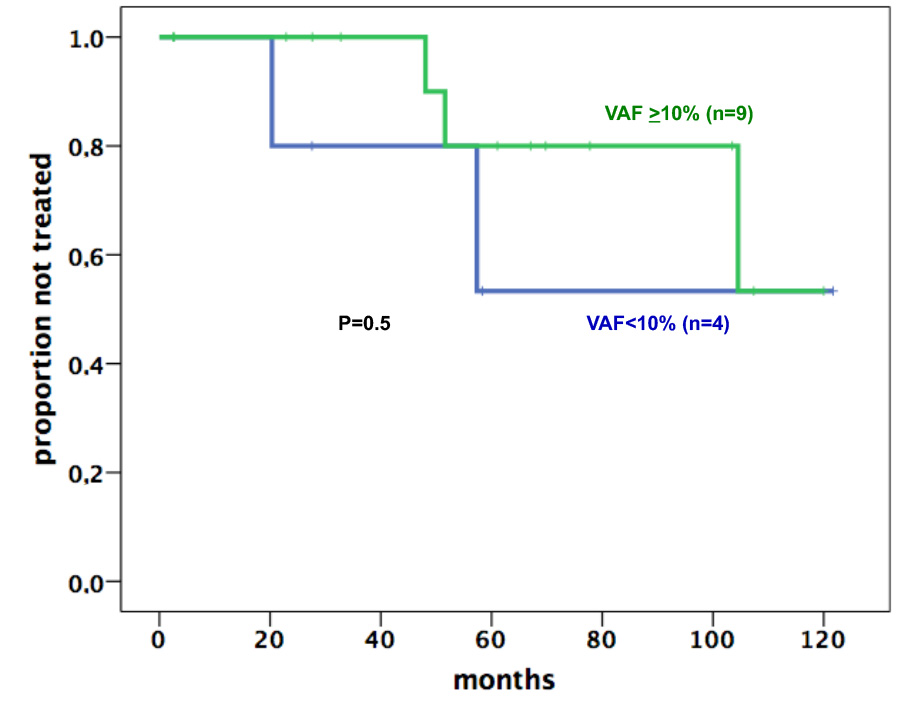


**Supplementary Figure 1.**

Clinical impact of the *TP53* mutations with different VAF, focusing on the M-IGHV subgroup of Mut/noDel patients. TTFT of patients with VAF<10% (n=4) and with VAF >10% (n=9) revealed no significant difference (P=0.5).

**Supplementary Figure 2**. **A)** Example of the functional heterogeneity of P53 mutant proteins in yLFM-P21-5, yLFM-PUMA, yLFM-MDM2P2C, and yLFM-BAX A+B yeast strains. The transactivation ability was determined using an inducible expression of P53 proteins (GAL1,10 promoter). Wild-type (WT) and mutant P53 fold induction over empty vector (pRS314) is used to calculate the transactivation ability (%) of the P53 mutant protein with respect to the WT. Presented is the average transactivation observed with the standard deviation of four biological replicates. **B)** Example of functional heterogeneity of P53 mutant proteins expressed in HCT116 *TP53*-/- cells transiently transfected with the corresponding expression vectors. WT and mutant P53 fold induction over empty vector (pCIneo) are used to calculate the transactivation ability (%) of the P53 mutant protein with respect to the WT. Presented is the average transactivation observed with standard deviation of four biological replicates. The last two colums for each reported strain and the last three columns in Panel A and B, respectively, represent the activity of the *TP53* germline allelic variants identified in our cohort (p.Asn235Ser, p.Arg283Cys, and p.Gly360Val).
